# Antibacterial and Antioxidant Activities of Derriobtusone A Isolated from *Lonchocarpus obtusus*

**DOI:** 10.1155/2014/248656

**Published:** 2014-06-01

**Authors:** Mayron Alves Vasconcelos, Francisco Vassiliepe Sousa Arruda, Daniel Barroso de Alencar, Silvana Saker-Sampaio, Maria Rose Jane Ribeiro Albuquerque, Hélcio Silva dos Santos, Paulo Nogueira Bandeira, Otília Deusdênia Loiola Pessoa, Benildo Sousa Cavada, Mariana Henriques, Maria Olivia Pereira, Edson Holanda Teixeira

**Affiliations:** ^1^Laboratory of Biologically Active Molecules, Biochemistry and Molecular Biology Department, Federal University of Ceará, 60.440-970 Fortaleza, CE, Brazil; ^2^Institute for Biotechnology and Bioengineering (IBB), Centre of Biological Engineering, University of Minho, 4710-057 Braga, Portugal; ^3^Integrated Laboratory of Biomolecules (LIBS), Department of Pathology and Legal Medicine, Federal University of Ceará, 62.042-280 Fortaleza, CE, Brazil; ^4^Marine Natural Products Laboratory, Department of Fishing Engineering, Federal University of Ceará, 60.440-970 Fortaleza, CE, Brazil; ^5^Centre of Exact Sciences and Technology, Acaraú Valley State University, 62.040-370 Sobral, CE, Brazil; ^6^Department of Organic and Inorganic Chemistry, Federal University of Ceará, 60.021-940 Fortaleza, CE, Brazil

## Abstract

This study evaluated the effect of derriobtusone A, a flavonoid isolated from *Lonchocarpus obtusus*, on two important pathogenic bacteria, *Staphylococcus aureus* and *Escherichia coli*, as well as its antioxidant activity and toxicity. Planktonic growth assays were performed, and the inhibition of biofilm formation was evaluated. In addition, antioxidant activity was assessed by DPPH radical scavenging assay, ferrous ion chelating assay, ferric-reducing antioxidant power assay, and **β**-carotene bleaching assay. Toxicity was evaluated by the brine shrimp lethality test. Results showed that derriobtusone A completely inhibited the planktonic growth of *S. aureus* at 250 and 500 **μ**g/mL; however, it did not have the same activity on *E. coli*. Derriobtusone A reduced the biomass and colony-forming unit (cfu) of *S. aureus* biofilm at concentrations of 250 and 500 **μ**g/mL. In various concentrations, it reduced the biofilm biomass of *E. coli*, and, in all concentrations, it weakly reduced the cfu. Derriobtusone A showed highly efficient antioxidant ability in scavenging DPPH radical and inhibiting **β**-carotene oxidation. The compound showed no lethality to *Artemia* sp. nauplii. In conclusion, derriobtusone A may be an effective molecule against *S. aureus* and its biofilm, as well as a potential antioxidant compound with no toxicity.

## 1. Introduction

Biofilm is a complex agglomeration of microbes adhering to a solid surface and to one another, all encased in a scaffold of self-produced extracellular polymeric substances [1]. By the ability to produce such extracellular polymeric substances, bacteria present in microbial biofilm show a reduced growth pattern, with up- and downregulation of specific genes [2]. Physiological and phenotypical adaptations that result in antimicrobial tolerance have been attributed to biofilm formation [3]. Biofilm formation is directly related to various infectious diseases through colonization on medical devices [4, 5]. Several pathogenic bacteria are capable of forming biofilms; among them are* Staphylococcus aureus* and* Escherichia coli* [6, 7]. However, plants are rich in a wide variety of molecules with antimicrobial properties, such as secondary metabolites and proteins [8]. In fact, several studies have reported on the antimicrobial and antibiofilm activities of plant compounds as alternatives to antibiotic therapy [9–12].

Furthermore, the antioxidant activities on reactive oxygen species (ROS) and other free radicals have been attributed to plant molecules, mostly phenolic compounds [13]. A common denominator of environmental stress is the production and accumulation of ROS, such as superoxide anions (O_2_^−^), hydrogen peroxide (H_2_O_2_^∙^), hydroxyl radicals (OH^∙^), and singlet oxygen (^1^O_2_) [13]. ROS accumulation leads to oxidative stress that can damage cellular components, such as DNA, lipids, proteins, and sugars [14, 15]. Moreover, ROS are associated with toxic effects and pathologies such as cancers, cardiovascular and neurological diseases, and infections [16]. In this context, the use of antioxidant compounds with the aim of increasing the degradation of ROS and thereby reducing ROS-associated diseases has been studied [17].

Some studies have reported that plants of the genus* Lonchocarpus* are able to produce compounds, such as alkaloids and triterpenoids, derived from benzoic acids and flavonoids [18–22].* Lonchocarpus* is a genus of the family Leguminosae, subfamily Papilionoideae, and it is prevalent in tropical and subtropical regions, including Brazil [20, 22].

Flavonoids are phenolic compounds consisting of two benzene rings linked through a heterocyclic pyrimidine ring [23]. Moreover, flavonoids have been reported to possess many useful properties, such as anti-inflammatory, antiallergic, antitumor, antioxidant, and antimicrobial activities [24–29]. Aurone constitutes a subclass of flavonoids consisting of a benzofuranone ring linked through a carbon-carbon double bond to a phenyl moiety [30], and auronol is an aurone derivative in which the benzylidene unsaturation has undergone hydration [31]. These compounds comprise a very small group of flavonoids [31, 32].

Derriobtusone A is a methylated auronol with a fairly rare occurrence (2-Benzoyl-3-methoxybenzo[1,2-b:3,4-b′]difuran.) [19]. This compound was the first auronol found in nature and was initially extracted and isolated from the roots of* Lonchocarpus obtusus* by Nascimento and colleagues [19, 20]. Furthermore, derriobtusone A also was isolated from the roots of* Lonchocarpus montanus*, being among the most abundant compounds found in this species [19]. Over thirty years have passed since derriobtusone A was identified. However, only few studies have evaluated the biological activities of this compound. Therefore, the present study aims to evaluate the antibacterial effects of derriobtusone A on* Staphylococcus aureus* and* Escherichia coli*. The antioxidant effects and toxicity of this compound were also demonstrated.

## 2. Material and Methods 

### 2.1. Plant Material

The roots of* Lonchocarpus obtusus* were collected from Meruoca City (Ceará State, Brazil). Plant authentication was performed by Professor Afrânio Gomes Fernandes, and a voucher specimen (number 39550) was deposited at the Herbário Prisco Bezerra (EAC) of the Departamento de Biologia, Universidade Federal do Ceará.

### 2.2. Derriobtusone A Isolation

**Figure 1:**
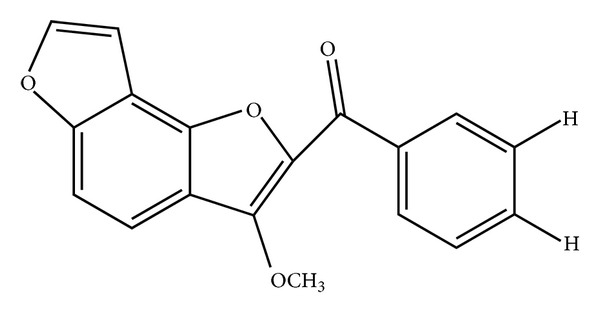
Chemical structure of derriobtusone A extracted from the root bark of* Lonchocarpus obtusus*.

Derriobtusone A was isolated as described previously by Cavalcante et al. [22]. Dried root bark (720 g) and wood (750 g) of* Lonchocarpus obtusus* were powdered and then extracted at room temperature with n-hexane (3 × 2.0 L). During the distillation process, a yellowish precipitate was filtrated, and the compound derriobtusone A was purified by crystallization in acetone (Figure 1).

### 2.3. Microorganisms

In the present study, the microorganisms used in the experiments were* Staphylococcus aureus* JKD 6008, a Gram-positive bacterium, and* Escherichia coli* ATCC 47076, a Gram-negative bacterium.

### 2.4. Culture Conditions

The bacteria were grown in Trypticase Soy Agar medium (TSA; Liofilchem, Italy) and incubated at 37°C for 24 h. After growth on the solid medium, an isolated colony was removed and inoculated into 10 mL of Trypticase Soy Broth (TSB; Liofilchem, Italy) and incubated for 18 h at 37°C under constant agitation of 120 rpm. Prior to use, the cell concentration of each inoculum was adjusted to 2 × 10^6^ cells/mL using a spectrophotometer (620 nm) and calibration curves previously determined for each bacterium.

### 2.5. Planktonic Growth Assay

The effect of derriobtusone A on planktonic growth of* S. aureus* and* E. coli* was determined by the broth microdilution method. Briefly, in 96-well polystyrene plates, derriobtusone A was diluted in TSB (with 4 % of dimethyl sulfoxide [DMSO]) in concentrations of 3.9 to 250 *μ*g/mL. The plates were incubated at 37°C during 24 h under constant agitation at 120 rpm. Optical density of the contents of each well was recorded at 640 nm (OD_640_) using an automated Elisa reader (Synergy TM HT multidetection microtiter reader), as a measure of microbial growth. Minimum inhibitory concentration (MIC) was established as the lowest concentration of compound able to inhibit the visible growth of microorganism after overnight incubation.

### 2.6. Biofilm Assays

The methodology used to grow biofilms was based on the microtiter plate test developed by Stepanovi$\overset{´}{\text{c}}$ et al. [33] with some modifications. Sterile 96-well polypropylene plates were prepared using a procedure similar to that used for the planktonic growth assays with the same initial concentration of cells and derriobtusone A. All plates were incubated on a horizontal shaker (120 rpm) at 37°C during 24 h for biofilm development. After biofilm growth in the presence or absence of derriobtusone A, the content of each well was removed, and the biofilms were washed twice with 200 *μ*L/well of sterilized water to remove weakly adherent cells.

#### 2.6.1. Biomass Quantification

Quantification of biofilm biomass was determined by crystal violet staining. For fixation of biofilms, 200 *μ*L of 99% methanol (Romil, UK) was added to each well, and, after 15 min, the methanol was removed and the plates were dried at room temperature. Then, 200 *μ*L of crystal violet stain (Merck, Germany) was added to each well. After 5 min, the excess of crystal violet was removed, and the plates were washed in water. Finally, 200 *μ*L of acetic acid (33%, v/v) (Pronalab, Portugal) was added to all wells to dissolve the crystal violet staining, and the absorbance was measured at 570 nm (OD_570_).

#### 2.6.2. Quantification of Colony-Forming Units

After biofilm formation, 200 *μ*L of sterile water was added to each well, and the plate was placed in an ultrasonic bath (Sonicor SC-52; Sonicor Instruments, Copiague, NY, USA, operating at 50 kHz during 6 min). Serial decimal dilutions from the obtained suspension were plated on TSA. The plates were then incubated for 24 h at 37°C, and the total number of colony-forming units (cfu) per unit area (log_10_cfu/cm^2^) of microtiter plate well was enumerated.

### 2.7. Antioxidant Assays 

#### 2.7.1. DPPH (1,1-Difenil-2-picrilidrazil) Radical Scavenging Assay

The DPPH scavenging activity of derriobtusone A at concentrations from 7.8 to 500 *μ*g/mL was measured according to the method described by Duan et al. [34]. The absorbance of sample, blank sample, and control was measured at 517 nm, after 30 min incubation in the dark at room temperature, using a Biochrom Asys UVM 340 microplate reader (Cambridge, UK). The sample consisted of a mixture of 1 mL DPPH methanolic solution (0.16 mM) with 1 mL of derriobtusone A. The blank sample consisted of 2 mL of derriobtusone A, while the control contained 2 mL DPPH methanolic solution (0.16 mM) only. Ascorbic acid was used as positive control. The percentage of DPPH scavenging activity was calculated with the following equation:

(1) Scavenging  effect(%)  =[1−(O.Dsample−O.Dblank)O.Dcontrol]×100%.

#### 2.7.2. Ferrous Ion Chelating (FIC) Assay

The ferrous ion chelating (FIC) power of derriobtusone A was determined with the method described by Wang et al. [35]. Distilled water, 2 mM ferrous chloride (FeCl_2_), and 5 mM ferrozine were added to the compound at concentrations from 7.8 to 500 *μ*g/mL. The blank sample and the control were prepared with distilled water instead of ferrozine and the compound, respectively. The sample, the blank sample, and the control were incubated at room temperature for 10 min, and the optical density was measured at 562 nm using a microplate reader (Biochrom Asys UVM 340). Ethylenediamine tetraacetic acid (EDTA) was used as positive control. FIC activity was calculated with the following equation:

(2) Ferrous  ion  chelating  activity(%)  =[O.Dcontrol−(O.Dsample−O.Dblank)]O.Dcontrol×100%.

#### 2.7.3. Ferric-Reducing Antioxidant Power (FRAP)

Ferric-reducing antioxidant power (FRAP) of derriobtusone A was determined using the method described by Ganesan et al. [36]. Initially, 0.2 M phosphate buffer (pH 6.6) and 1% potassium ferricyanide were added to the compound at different concentrations (7.8 to 500 *μ*g/mL). The samples were then incubated at 50°C for 20 min. After cooling at room temperature, 10% trichloroacetic acid was added. An aliquot was mixed with distilled water and 0.1% ferric chloride. Ten minutes later, the optical density was measured at 700 nm using a microplate reader (Biochrom Asys UVM 340). Butylated hydroxyanisole (BHA) was used as positive control. Greater absorbance indicated greater FRAP.

#### 2.7.4. *β*-Carotene Bleaching (BCB) Assay

The coupled oxidation of *β*-carotene and linoleic acid was determined with the method described by Chew et al. [37]. Tween 40 was added to *β*-carotene (100 *μ*g/mL in chloroform) and linoleic acid. Following evaporation of the chloroform in a rotating evaporator, oxygen-saturated ultrapure water (Milli-Q) was added, and the mixture was shaken until forming an emulsion. Thus, the final sample was a mixture of emulsion and compound, while the control consisted of emulsion only. The optical density was measured at 470 nm using a microplate reader (Biochrom Asys UVM 340), followed by another 3 hours of incubation at 50°C. BHA was used as positive control. The antioxidant activity was calculated with the following equation:

(3) $$\begin{array}{c} \text{Antioxidant}\;\text{activity}\left(\%\right)=\left(\frac{\text{O}.{\text{D}}_{\text{final}}}{\text{O}.{\text{D}}_{\text{initial}}}\right)\times 100\%. \end{array}$$

### 2.8. *Artemia* Lethality Test

*Artemia *sp. cysts were hatched in sterile artificial seawater at 28°C under constant light and strong aeration. The cysts were incubated in a polyethylene cylindroconical tube with 1 g of cysts per liter of artificial seawater. After a period of 48 h, the nauplii were collected and used for bioassays.

Derriobtusone A was dissolved in artificial seawater (with 4% DMSO) at a concentration of 1 mg/mL. The assay was performed boarding 24-well Linbro plates in which each well contained 10* Artemia* sp. nauplii in a final volume of 2 mL. The compound solution was added to the wells at final concentrations of 7.8 to 500 *μ*g/mL. The experiments were performed in triplicate, and negative control wells contained 2 mL of artificial seawater (with 4% DMSO) with 10* Artemia* sp. nauplii. After 24 and 48 h, the number of dead nauplii in each well was counted.

### 2.9. Statistical Analysis

Statistical analyses were performed by GraphPad Prism version 5.0 from Microsoft Windows. The data from all the antimicrobial and biofilm assays were compared using one-way analysis of variance (ANOVA), with Bonferroni post hoc test. *P* < 0.05 was considered to be statistically significant.

For antioxidant assays, the percentage values obtained for DPPH, FIC, and BCB with each concentration were converted into absolute values, submitted to angular transformation, and compared with Student's* t-*test for independent data. FRAP values, as obtained for each concentration, were also analyzed with Student's* t-*test for independent data. *P* < 0.05 was considered to be statistically significant.

## 3. Results 

### 3.1. Effect of Derriobtusone A on Planktonic Growth

**Figure 2:**
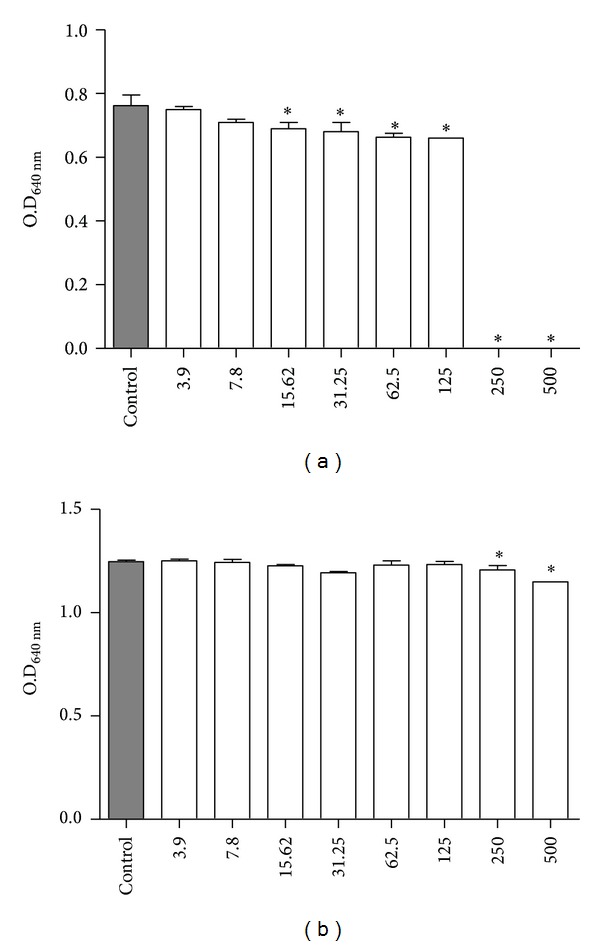
Effect of derriobtusone A on planktonic growth of (a)* S. aureus* and (b)* E. coli*. *P < 0.05 compared to control.

The results showed that derriobtusone A inhibited the planktonic growth of* Staphylococcus *aureus at concentrations of 250 (MIC) and 500 *μ*g/mL. When bacteria were treated with concentrations ranging from 15.5 to 125 *μ*g/mL, only a weakly inhibition was seen (Figure 2(a)). On the other hand, derriobtusone A at concentrations of 250 and 500 *μ*g/mL showed a weakly inhibition on* Escherichia coli* planktonic growth (Figure 2(b)).

### 3.2. Effect of Derriobtusone A on Biofilm

**Figure 3:**
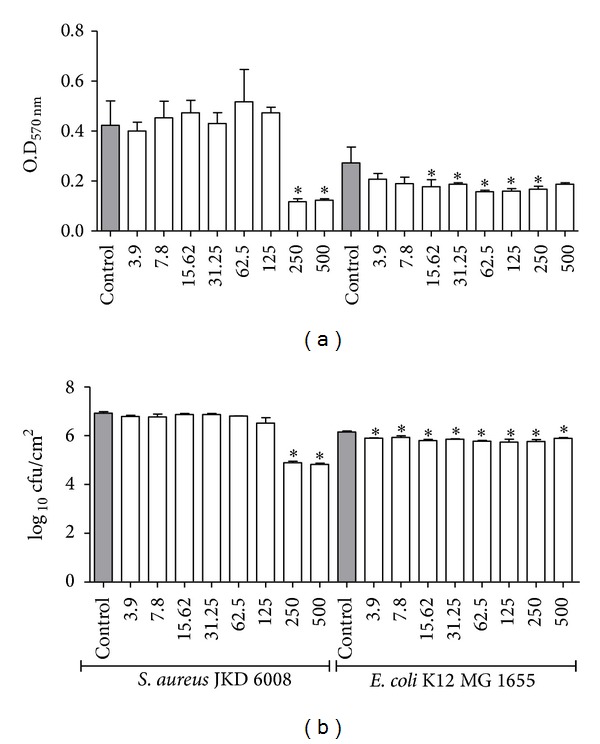
Effect of derriobtusone A on biofilm of* S. aureus* and* E. coli*. (a) Biofilm biomass and (b) enumeration of cfu. *P < 0.05 compared to control.

Figure 3(a) presents the results of biofilm biomass of* Staphylococcus aureus* and* Escherichia coli* after 24 h of contact with derriobtusone A. At concentrations of 250 and 500 *μ*g/mL, data showed that the compound was able to abruptly reduce the biomass of* Staphylococcus aureus* and significantly reduce the biomass of* Escherichia coli* at concentrations ranging from 15.6 to 250 *μ*g/mL.

Figure 3(b) presents mean values and standard deviations of log_10_cfu/cm^2^ for both* Staphylococcus aureus* and* Escherichia coli*. Similar to the biomass results, derriobtusone A at concentrations of 250 and 500 *μ*g/mL reduced the cfu in* S. aureus* biofilm by approximately 2.0 log_10_. Interestingly, derriobtusone A was able to reduce the cfu in* Escherichia coli *biofilm between 0.2 and 0.4 log_10_ in all concentrations.

### 3.3. Antioxidant Activity of Derriobtusone A

Derriobtusone A antioxidant activity was evaluated by four different methods: DPPH radical scavenging, FIC, FRAP, and BCB.

**Figure 4:**
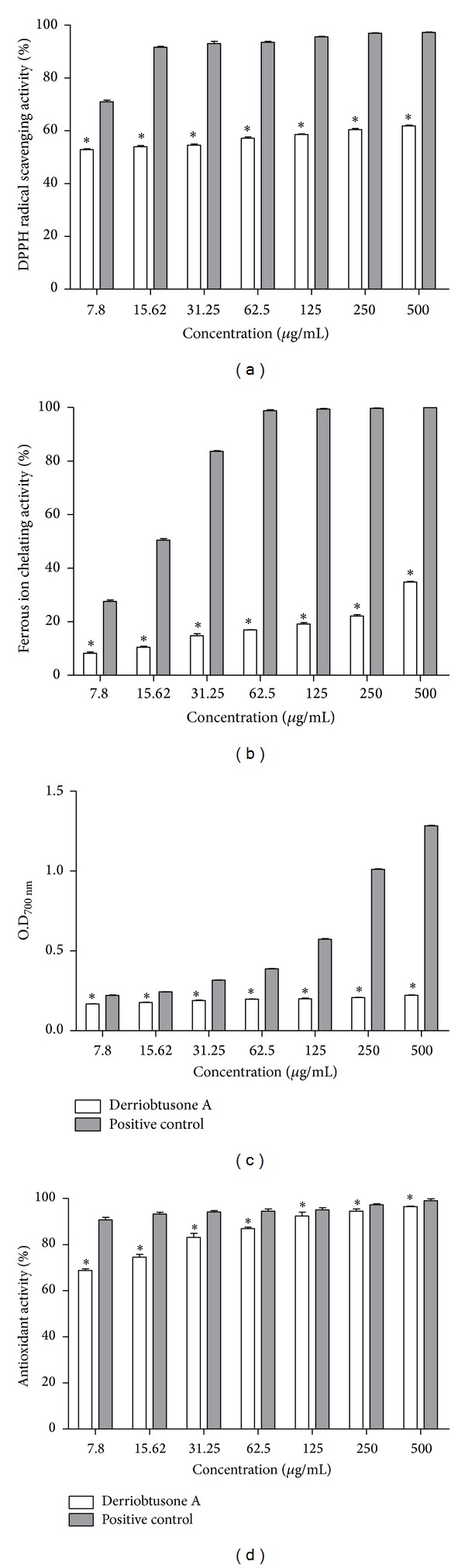
Antioxidant activity of derriobtusone A. (a) Scavenging activity of DPPH (%), (b) chelating ability of ferrous ion (%), (c) reducing power of ferric ions, and (d) *β*-carotene bleaching assay (%).*P < 0.05 compared to positive control.

In the DPPH assay, the compound was able to scavenge DPPH radical in all concentrations, performing this activity between 50 and 60%. Ascorbic acid (positive control) showed significant difference compared to derriobtusone A in all concentrations, performing this activity between 70 and 97% (Figure 4(a)).

Using the FIC assay, derriobtusone A showed chelating ability of ferrous ion by approximately 8, 10, 14, 17, 19, 22, and 34 % in concentrations of 7.8 to 500 *μ*g/mL, respectively, while the positive control (EDTA) showed significantly greater activity than the compound. In the higher concentrations, EDTA showed approximately 100% activity (Figure 4(b)).

Derriobtusone A was evaluated by ferric reducing/antioxidant power assay to determine its ability to reduce Fe^3+^ to Fe^2+^. In this assay, the results showed that optical density increased weakly with increasing concentrations of the compound. The variation of optical density for derriobtusone A ranged from 0.168 to 0.222, while BHA, the positive control, showed an abrupt increase of 0.226 to 1.283 in optical density (Figure 4(c)).

The ability of derriobtusone A to inhibit *β*-carotene oxidation was also evaluated. The compound showed a high antioxidant activity, ranging from 68.7 to 96.5%. Moreover, the antioxidant activity was similar to positive control in the higher concentrations (Figure 4(d)).

### 3.4. Toxicity of Derriobtusone A on* Artemia* sp

The toxicity of the derriobtusone A on* Artemia* sp. nauplii was evaluated after 24 and 48 hours of exposure. Interestingly, the compound showed no lethality to* Artemia* sp. nauplii at any of the concentrations tested (data not shown).

## 4. Discussion

*Staphylococcus aureus* and* Escherichia coli *are two microorganisms generally associated with human infections, and they exhibit considerable antimicrobial resistance based on the ability to form biofilms [6, 7, 38, 39]. This study evaluated the effect of derriobtusone A, a flavonoid isolated from* Lonchocarpus obtusus*, on planktonic growth and biofilm formation of* Staphylococcus aureus* and* Escherichia coli*.

The planktonic growth assay showed that* Staphylococcus aureus* was susceptible to the presence of derriobtusone A in that the molecule totally inhibited bacterial growth at 250 *μ*g/mL. On the other hand,* Escherichia coli* showed more resistance against the compound. This study corroborated the findings of Magalhães et al. [40] who showed that derriobtusone A isolated from* Lonchocarpus montanus *was activeagainst* Staphylococcus aureus*, but not against* Escherichia coli *in a bioautography assay. Other studies have also shown the antibacterial activity of flavonoids in different concentrations [28, 29]. Panduratin A, a chalcone isolated from* Kaempferia pandurata*, showed antibacterial action against clinical* Staphylococcus* strains in concentrations between 0.063 and 2 *μ*g/mL [41]. Sepicanin A, a flavanone isolated from* Artocarpus sepicanus*, inhibited the bacterial growth of* Staphylococcus aureus* at 1.2 *μ*g/mL [42]. Furthermore, the molecule 4′-methoxyflavanone showed action against* Staphylococcus aureus* in concentrations up to 1000 *μ*g/mL [43].

The differences in antibacterial activity of flavonoids at various concentrations have also been investigated. For example, according to Basile and coworkers [44], apigenin, a flavanone isolated from* Castanea sativa*, inhibited the growth of* Staphylococcus aureus* at concentrations up to 128 *μ*g/mL, but, in another study, Sato and coworkers [45] showed that this same flavonoid isolated from* Scutellaria barbata* inhibited the growth of strains of* Staphylococcus aureus *at concentrations between 3.9 and 15.6 *μ*g/mL. These discrepancies could be attributed to the different techniques employed to evaluate the antimicrobial action of flavonoids, different solvents used to dissolve the molecules, or the provenance of flavonoid [29].

Flavonoids are diverse compounds of plants which play important roles in growth and defense against microorganisms and pests [46]. In fact, the antibacterial activity of flavonoids is linked to their structure. Li and colleagues [47] showed that the linking of N-heterocyclic ring to the A ring of chrysin makes this molecule 16- to 32-fold more active against* Escherichia coli* and* Staphylococcus aureus* than its parent compound. Several antibacterial mechanisms of action have been assigned to these compounds. According to Cushnie and Lamb [29], the possible mechanisms of action of the flavonoids are as follows: damage in cytoplasmic membrane, causing pores in membrane or reduction in fluidity; inhibition of nucleic acid synthesis by the inhibition of the enzyme topoisomerase; inhibition of cellular metabolism, resulting from inhibition of the enzyme NADH-cytochrome C reductase; inhibition of cell wall synthesis caused by D-alanine/D-alanine ligase inhibition; inhibition of cell membrane synthesis; and aggregation of bacterial cells. Furthermore, some studies suggest that flavonoids have multiple mechanisms of action [29, 48, 49].

This study showed that derriobtusone A inhibited biofilm formation, reducing the biomass and number of viable cells of* Staphylococcus aureus* (Figure 3) at concentrations of 250 and 500 *μ*g/mL. Interestingly, although the planktonic growth of* Escherichia coli* was only weakly inhibited by derriobtusone A, the compound did reduce biomass in various concentrations and did weakly reduce the number of viable cells. Although several studies have reported on the antibacterial action of flavonoids, few works describe the potential antibiofilm activity of these molecules. A synthetic flavonoid, 3-arylideneflavanone 2C, showed antibacterial activity against* Staphylococcus aureus* and inhibited the initial adhesion of bacteria to abiotic surfaces, resulting in blocking biofilm formation [50]. Vikram and colleagues [46] showed that citrus flavonoids were able to reduce the biofilm formation of* Vibrio harveyi* and* Escherichia coli*. Using lectins, Liljemark and colleagues [51] showed that the formation of cellular aggregates decreased the number of adherent streptococci and, consequently, biofilm formation. Flavonoids have the ability to aggregate bacterial cells, thereby potentially explaining the antibiofilm activity of derriobtusone A [29]. Furthermore, an apple flavonoid, phloretin, inhibits suppressed autoinducer-2 importer genes (*lsrACDBF*) of* Escherichia coli*, suggesting that this compound interferes with bacterial quorum-sensing (AI-2) signaling. This study also showed that phloretin reduces fimbria production of* Escherichia coli* [52]. Fimbriae are important structures that influence bacterial adhesion. Thus, the inhibition of fimbria formation could be a way to decrease biofilm formation [52, 53].

In addition, derriobtusone A showed potential antioxidant activity (Figure 4). Plant phenolic compounds, such flavonoids, have been associated with the health benefits derived from consuming high levels of fruits and vegetables, and these effects have been attributed to their antioxidant activity [54, 55]. According to Narsinghani and colleagues [56], aurone derivatives have displayed several pharmacological activities, including antioxidant activities, and changes in their structure have been important in the development of new antioxidant compounds with improved potency and less toxicity.

In the present study, derriobtusone A demonstrated its ability to scavenge DPPH radical. Even at the lowest concentration tested, the compound displayed over 50% DPPH activity and also showed moderate chelating ability of ferrous ion. These results corroborate those of Narsinghani and colleagues [56] who showed significant antioxidant activity for five synthetic aurones, using these same two methods; however, the synthetic compounds showed a satisfactory reducing power, a fact not evidenced in this study. Moreover, the compound showed a high potential for inhibiting *β*-carotene oxidation by protecting it against free radicals generated during linoleic acid peroxidation, in some concentrations with activity similar to that of BHA. It is interesting to note that both derriobtusone A and BHA are compounds that exhibit apolar characteristics. Furthermore, according to Frankel and Meyer [57], antioxidants with apolar properties are most important because they are concentrated in the lipid-water interface, thereby preventing the formation of lipid radicals and *β*-carotene oxidation. Another interesting observation is that BHA at high concentrations may induce gastrointestinal hyperplasia [58].

Studies have shown that the antioxidant activity of flavonoids depends on the arrangement of functional groups about the nuclear structure [54, 59]. Multiple hydroxyl groups and their position in the molecule confer upon the molecule substantial antioxidant, chelating, and prooxidant activity, while* O*-methylation can suppress the action of the compound [60, 61]. Other structural features important for the antioxidant activity of flavonoids are the presence of unsaturation in conjugation with a 4-oxo-function in the C-ring and the presence of functional groups capable of binding transition metal ions. The lack of these groups in derriobtusone A may explain the moderate chelating ability of ferrous ion and ferric-reducing power presented in this study [62].

The question of flavonoid toxicity has been raised, even though this compound is widely distributed in edible plants and beverages [28]. Despite the minimal toxicity in such plants and beverages, Magalhães and colleagues [40] showed that flavonoids from* Lonchocarpus montanus* displayed high toxicity in their brine shrimp lethality assay, including derriobtusone A (LD_50_ − 1.6 *μ*g/mL). Corroborating this result, Santos and colleagues [21] showed that the extract from the root bark of* Lonchocarpus filipes *and three flavonoids isolated from this plant displayed high toxicity using the same assay. Interestingly, derriobtusone A from* Lonchocarpus obtusus* was shown to be nontoxic to* Artemia *sp. nauplii in the present study.

In summary, derriobtusone A showed potential antibacterial activity against* Staphylococcus aureus *and the ability to inhibit its biofilm formation. A decrease of* Escherichia coli* biofilm biomass was also shown. Furthermore, the compound showed antioxidant potential without toxicity. Thus, derriobtusone A may be a potential agent against infections caused by* Staphylococcus aureus* biofilms, and it may also have antioxidant properties for possible use against oxidative stress.
